# DUX4_HD2_-DNA_ERG_ structure reveals new insight into DUX4-Responsive-Element

**DOI:** 10.1038/s41375-018-0273-z

**Published:** 2018-10-12

**Authors:** Xue Dong, Hao Zhang, Nuo Cheng, Kening Li, Guoyu Meng

**Affiliations:** State Key Laboratory of Medical Genomics, Shanghai Institute of Hematology, Rui-Jin Hospital, Shanghai JiaoTong University School of Medicine and School of Life Sciences and Biotechnology, Shanghai JiaoTong University, 197 Ruijin Er Road, Shanghai, 200025 China

**Keywords:** Acute lymphocytic leukaemia, Biochemistry

Recently, we have reported a 2.6 Å crystal structure of DUX4_HD2_ complexed with a consensus DRE_consensus_ derived from the wild type DUX4 ChIP-seq analysis [1, 2] The DRE_consensus_ site is also present in the leukemia NALM6 and Reh cells harboring oncogenic DUX4/IGHs (Fig. 1a and Supplementary Figure 1a) [3, 4]. Furthermore, the GATXXGAT-like, TGAT-ATTA-like repeats are also frequently associated with wild type DUX4 and DUX4/IGH target genes (Fig. 1a). In order to gain more insight into the true nature of DUX4-DRE interaction, we have determined the structure of DUX4 HD2 bound with endogeneous ERG DNA sequences derived from the B-ALL patient RNA-seq and ChIP-seq analysis [3, 4].

**Fig. 1:**
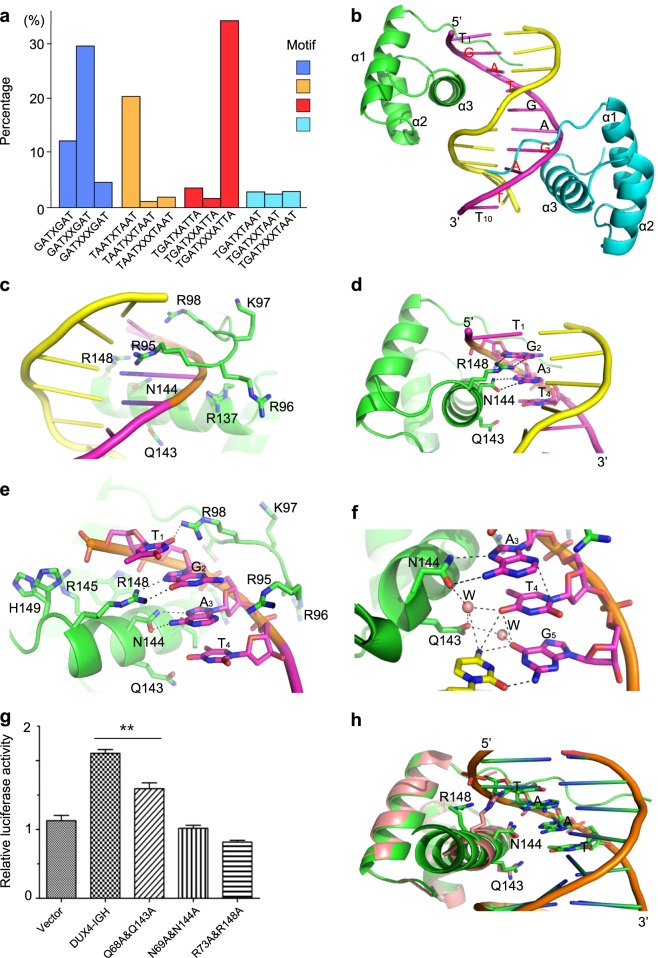
Structural and functional cauterization of DUX4-Responsive-Element (DRE) sites. **a** Repetitive motifs in wild type DUX4 and DUX4/IGH target genes. The published DUX4 and DUX4/IGH ChIP-seq datasets were used for this re-analysis. The percentage of different TGAT- and TAAT-like repeats in the DUX4 and DUX4/IGH target genes are shown in this figure. **b** ERG DNA duplex in complex with two HD2s. The ERG chains are colored with magenta and yellow. The 5′ and 3′ ends are labeled. The forward chain of 5′-T_1_GATGAGATT_10_-3′ is labeled. The repetitive GAT sequences are highlighted in red. Of note, nucleotide A11 is disordered and absence in the final model. HD2 molecules are colored with green and cyan, respectively. **c** Enlarged view of minor groove engagement by poly-Arg/Lys motif. The protein side-chains are labeled and shown in stick representation. **d** Enlarged view of major groove interaction by QNR motif. The DNA nucleotide T_1_GAT_4_ (magenta) are labeled and shown in stick representation. The hydrogen bonds are shown in dashed lines. **e** Other DNA-binding residues around T_1_GAT_4_. A highly positive pocket delineated by residues Arg95, Arg96, Lys97, Arg98, Arg137, Trp141, Arg145, Arg148 and His149 are assembled upon DNA binding. **f** The water-mediated hydrogen network around Gln143. Water molecules observed in between Gln143 and T_4_G_5_ nucleotide are shown in sphere representation. The hydrogen-bonding network are shown in dashed lines. **g** Luciferase assay using ERG site. The DRE site derived from *ERG* gene [4] was used in this assay. The transcriptional activities of the wild type DUX4/IGH and the mutations that target the QNR motifs in HD1-HD2 were characterized by the standard luciferase reporter kit (Promega). The luciferase activities were presents as mean ± SE. The statistical significance was assessed by the student’s *t* test analysis (**P < 0.01). **h** Structural superimposition between DUX4 (pink) and PAX (green) HDs in the context of PAX-TAAT structrue (PDB code: 1FJL). The different QNR motifs are shown in stick representation. T_1_AAT_4_ are labeled

The recombinant DUX4 HD2 domain was purified as described before [1]. The crystal of HD2-DRE_ERG_ diffracted remarkably well (1.6 Å) compared with that of DUX4_HD2_–DRE_consensus_ (2.6 Å). The statistics detail of X-ray data collection is shown in Table 1. For structural determination, the refined HD2 structures (PDB codes: 5Z2S and 5Z2T) [1], but not DNA coordinates, were used for molecular replacement (MR) approach implemented in PHASER [5]. The DNA duplex of 5′-TGATGAGATTA-3′/3′-ACTACTCTAAT-5′ were built manually using COOT [5], followed by TLS refinement using PHENIX.REFINE [6]. The final *R* and *R*_free_ factors are 20.1 and 20.7%, respectively.

**Table 1:** Data collection and structure refinement statistics of DUX4 HD2 associated with ERG site

Protein	DUX4_HD2_-DNA_ERG_
*Data collection*	
Space group	*P*3_1_
Unit cell dimension (Å)	
a	32.6
b	32.6
c	126.6
Molecule per ASU	2HD2, 1 double-chain DNA_ERG_
Derivative	Native
Source/Station^a^	BL19U
Wavelength (Å)	0.9785
Resolution range (Å)	63.3–1.60
Observations (*I* / σ(*I*) > 0)	160629
Unique reflections (*I* / *σ*(*I*) > 0)	19696 (2803)
High-resolution shell (Å)	1.69–1.60
*R*_sym_ (%)^b,c^:	7.2 (91.2)
<*I* / σ(*I*)>^c^:	11.4 (1.8)
Completeness^c^ (%):	99.2 (97.0)
Redundancy^c^:	8.2 (6.8)
CC_1/2_	0.99 (0.76)
*Structure refinement*	
Resolution range (Å)	28.2–1.60
*R*-factor (%)	20.1
*R*-factor (high-resolution shell)^d^	28.3
*R*_free_ (%)^e^	20.7
*R*_free_ (high-resolution shell)	34.7
Total number of non-hydrogen atoms	1492
Protein atoms	1376
Water molecules	116
R.m.s. deviations:^f^	
Bond length (Å)	0.013
Bond angle (°)	1.227
Main chain *B*-factors (Å^2^)	3.676
Side-chain *B*-factors (Å^2^)	7.047
Wilson *B*-factor (Å^2^)	28.4
Average *B*-factor protein atoms (Å^2^)	40.3
Ramachandran statistics (%)	
Most favored region	96.4
Allowed regions	3.6
Outliers	0

^a^Beamline designations refer to the Shanghai Synchrotron Radiation Facility, Shanghai, P. R. of China

^b^*R*_sym_ = Σ(*I*- < *I* > )^2^/Σ*I*^2^

^c^Overall, high-resolution shell in parentheses

^d^High-resolution shell: 1.6847–1.6000

^e^*R*_free_ calculated using 5% of total reflections omitted from refinement

^f^R.m.s. deviations report root mean square deviations from ideal bond lengths/angles and of *B*-factors between bonded atoms [8]

Consistent with previous report [1], one ERG DNA duplex can bind to two HD2 molecules (Fig. 1b). Unlike the previous 2.6 Å HD2-DRE_consensue_ structure, the electron density map of ERG DNA is of high quality (1.6 Å, Supplementary Figure 1b) and allows clear registration of ERG sequences, 5′-T_1_GATGAGATT_11_-3′/3′-A_1_CTACTCTAA_11_-5′. The electron density map of the last pair of nucleotides, T11 and A11, are disordered and hence not available for model building. For the HD2 molecules, the final refined models contain residues Arg95 to Gln152. As reported before [1], the HD2 domain folds into a global domain of three helices, α1–α3, respectively. The N-terminal poly-Arg/Lys motif, perpendicular to the helix α1, engages the DNA binding. In this structure, the Arg95 and Arg98 side-chains dip into the minor groove. In particular, Arg98 forms a hydrogen bond with the hydroxyl group of T1 nucleotide (Fig. 1c). Consistent with previous observation [1], the average *B* factor of R_95_RKR_98_ in DUX4_HD2_-DNA_ERG_ is 67.4 Å^2^, much higher than the rest of the structure (40.3 Å^2^), reiterating a secondary role in the two-step mechanism of DUX4-driven transactivation [1].

In current HD2-DNA_ERG_ structure, the QNR motif is also the major DNA-code-reading module (Figure 1d–f). The previous report suggests QNR can bind to the consensus TAAT repeat [1]. To our surprise, the Asn144 and Arg148 form two pairs of hydrogen bond with the G2 and A3 nucleotide (Fig. 1d). The invariant Asn144 among homeobox superfamily lies in the heart of the major groove. The carboxamide side-chain form two hydrogen bonds with the A3 nucleotide (2.7 and 3.0 Å, respectively). In parallel with Asn144 side chain lies the Arg148 guanidinium head group, which in turn forms two hydrogen with the G2 nucleotide (3.1 and 3.1 Å, respectively). Besides, in the region surrounding T_1_GAT_4_ nucleotides, it is enriched with positively charged residues including Arg95, Arg96, Lys97, Arg98, Arg137, Trp141, Arg145, Arg148 and His149 (Fig. 1e). Of note, the dual side-chain configuration observed in His149 is a strong indication of side-chain reshuffle upon DNA binding/recognition. In the major groove also lies another important residues, Gln143. Consistently with previous observation, the high-resolution DUX4_HD2_-DNA_ERG_ structure reveals an interesting Gln143-water-nucleotide network in the major groove (Fig. 1f). Furthermore, via water-mediated hydrogen-bonding network, Gln143 can contribute to side-chain orientation of Asn144 and its subsequent reading/binding of A3 nucleotdie.

The importance of QNR motif in DUX4/IGH-driven transactivation was vigorously checked in our previous report [1]. Here, the major DNA-reading motif was characterized with luciferase assay using ERG DNA sequence. The perturbation of the DNA engaging reisidues Gln68/Gln143, Asn69/Asn144 and Arg73/Arg148 significantly impaired the transcription activity of DUX4/IGH (Fig. 1g). In good agreement with our previous assays, the mutations of Asn69/Asn144 and Arg73/Arg148 were always more destructive when compared to that of Gln68/Gln143, reiterating the biological relevance of HD2-DNA_ERG_ and HD2-DRE_consensus_ structures. Indeed, the Asn144, Arg148 and Gln143 residues/positions appear to be the most, relative less and least conserved positions, respectively (Supplementary Figure 2). This has led to the proposal that, while the invariant Asn residue in the middle of the DNA-binding-triol might play the most critical role in DNA binding, the residues Gln/Arg (or other variants) in the flanking positions might contribute to motif recognition specificity (Supplementary Figure 2).

Until now, it is well recognized that most HD domains can bind DNA with TAAT motif. Like PAX/PAX3, DUX4 HDs, which contain the same sets of poly-Arg/Lys and QNR motifs, can interact with TAAT. Agreeably, using luciferase assay, Zhang and co-workers demonstrated that TAAT-rich sequences were required for DUX4-driven transactivation [7]. Consistently, TAAT repeat was also frequently associated with leukemia cell lines that contain DUX4/IGH [3, 4] (Fig. 1a and Supplementary Figure 1a).

In this report, the 1.6 Å DUX4_HD2_-DNA_ERG_ structure demonstrates a strong association between DUX4 homeobox and TGAT. As discussed in our previous report [1], the coordinates and positioning of the second HD2 molecule in this structure also allow the envisage/modeling of DUX4_HD1-HD2_ complexed with ERG site 5′-TGATGAGATTA-3′, in which it also contains repetitive GAT sequences. This has led to the re-examination of the previous model, HD2-DRE_consenesus_ (the consensus DNA sequence used in previous crystallization is 5′-TTCTAATCTAATCA-3′/3′-AAGATTAGATTAGT-5′). When GAT repeat was modeled into the DNA electron density map that engages QNR interaction, the *R*_*free*_ factor is 29.4% (previous refinement is 29.9%). In light of the new HD2-DNA_ERG_ high-resolution structure, the previous DNA registration in the poor electron density map of HD2-DRE_consensue_ (*R*_*sym*_ and signal-to-noise level in the highest resolution shell 2.7–2.6 Å is 155% and 1.5, respectively) might be interpreted with caution although it suggests a TAAT-binding possibility. In this model, it is clear that DUX4 HD2 can bind to GATXXGAT-like repeat. In support of this claim, the revision of the published DUX4/IGH ChIP-seq data showed that, among 364 DUX4 target genes, 109 genes contain GATXXGAT motif (Fig. 1a). In addition, TAAT-like repeat is also frequently reported in wild type DUX4 and DUX4/IGH [3, 7] (Fig. 1a). Consistently, via structural superimposition and sequence alignment, a possible engagement between DUX4 HDs and TAAT site could be envisaged (Fig. 1h). Indeed, the TGAT motif (in the forward DNA chain) and the TAAT motif (in the complementary DNA chain) display the strongest consensus in DUX4-driven transactivation (Supplementary Figure 1a). Taken together, the new DUX4_HD2_-DNA_ERG_ structure shed new insight into the DUX4-DRE recognition: (1) DUX4 HD1-HD2 might bind preferentially to the repetitive DNA sequences containing 5′-GATXXGAT-3′, 5′-TAATXTAAT-3′ and 5-TGAT/TAAT-5′ (the complementary chain is underlined) motifs. (2) This helps to define a novel HD subclass, in which DUX double homeobox can display remarkable double-kiss and double tolerance activities in recognition of TGAT- and TAAT-like repetitive sequences. In addition, the current observation/proposal in DUX4 will undoubtedly prompts a more extensive revision of the versatile HD-DNA interactions summarized in Supplmentary Figure 2.

## Electronic supplementary material

Supplementary Text

Supplementary Figure 1

Supplementary Figure 2
