# Supplementary material for: DUX4HD2-DNAERG structure reveals new insight into DUX4-Responsive-Element
Source: Leukemia. 2018 Oct 12;33(2):550–3. doi: 10.1038/s41375-018-0273-z (PMC6365376; doi:10.1038/s41375-018-0273-z)
Supplement: Supplementary file 1 — Supplementary Text [file 41375_2018_273_MOESM1_ESM.doc]

**DUX4_HD2_-DNA_ERG_ structure reveals new insight into DUX4-Responsive-Element**

Xue Dong^*^, Hao Zhang^*^, Nuo cheng^*^, Kening Li, Guoyu Meng^Ψ^

State Key Laboratory of Medical Genomics, Shanghai Institute of Hematology, Rui-Jin Hospital, Shanghai JiaoTong University School of Medicine and School of Life Sciences and Biotechnology, Shanghai JiaoTong University, 197 Ruijin Er Road, Shanghai 200025, China

**Running title: Structural characterization of DUX4-Responsive-Element**

^*^ equal contribution

^Ψ^Senior and corresponding authors

E-mail: [guoyumeng@shsmu.edu.cn](mailto:guoyumeng@shsmu.edu.cn)

Tel: 0086 (0) 2164370045-610730

Fax: 0086 (0) 2164743206

**Keywords**: acute lymphoblastic leukemia, DUX4/IGH, ERG binding site, GAT repeat

**Materials and methods**

***Protein and DNA preparation***

The DNA fragment encoding residues 94-153 of human DUX4 protein (i.e. HD2 domain) was cloned into a bacterial expression vector pET15b under the T7 promoter. The same expression and purification protocols described in our previous report were used to obtain recombinant DUX4 HD2. The correct mass of the target protein was confirmed by MS analysis, and the purity of >95% was assessed by SDS-PAGE.

Synthetic single stranded ERG DNA oligonucleotides of 5’-TGATGAGATTA-3’ and 3’-ACTACTCTAAT-5’ were re-suspended in sterile water, respectively. In order to obtain double stranded DNA, the oligonucleotides were mixed at a 1:1 molar ratio, then annealed under 95 °C for 10 min and slowly cooled to 4°C. The annealed ERG DNA was concentrated to a final concentration of 77 mg/ml measured by the absorbance at 260 nm.

***Crystallization, data collection and structural determination***

Crystallization experiments were carried out using the hanging drop vapor-diffusion method. For co-crystallization of the DUX4_HD2_-DNA_ERG_, the double-stranded DNA_ERG_ and DUX4_HD2_  protein (25 mg/ml) were incubated at a 1:1 molar ratio at 4 °C for 30 min prior to crystallization screen at 20°C. Diffracting crystals of DUX4_HD2_-DNA_ERG_ were obtained in the condition containing 100 mM Hepes (pH 7.5), 25% PEG1000.

The crystals were flash-cooled in liquid nitrogen using oil as cryoprotectant. The diffraction data were recorded in BL19U1 at Shanghai Synchrotron Radiation Facility (SSRF, Shanghai, China). The DUX4_HD2_DNA_ERG_ crystals diffracted to 1.6 Å and were in space group *P*3_1_. The diffraction data were processed, integrated and scaled using MOSFLM/SCALA[5]. The statistics of the data collection are shown in Table 1.

For DUX4_HD2_-DNA_ERG_ structural determination, the previous refined HD2 structures (PDB codes: 5Z2S and 5Z2T)[1], were used as search templates. Initially, *F*o-*F*c difference map was used to build the DNA nucleotides via intermittent manual building implemented in COOT[5]. PHENIX.REFINE[6] was used for structural refinement. The resulted 2*F*o-*F*c and *F*o-*F*c map were subsequently used to correct and improve the initial model. The *B*-factors were refined with TLS corrections. The final model of the asymmetry unit (ASU) contains one DNA duplex, two HD2 molecules and 116 water molecules. The coordinates and experimental diffraction data have been deposited into PDB database under the entry code of 6A8R. The detailed structure refinement statistics are reported in Table 1.

***Luciferase assay***

The DNA binding region of *ERG* was cloned into the pGL4.15 firefly luciferase reporter vector (Promega). The reporter construct, a Renilla luciferase vector used for control of transfection efficiency, and the wild type DUX4/IGH or its mutant plasmids were cotransfected into 293T cells using Lipofectamin 2000. Twenty-four hours after transfection, cells were harvested for the determination of luciferase activities using the Dual-luciferase reporter assay kit (Promega).

***ChIP-seq analysis***

The ChIP-seq data of normal human myoblasts (i.e. the wild type DUX4 dataset) and leukemia NALM6 and Reh cells (i.e. the DUX4/IGH dataset) were obtained from GEO database (GSE75791)[9] and EGA database (EGAS00001001923)[4]. Bedtools (v2.27.0)[10] was used to overlap the two datasets. The HOMER software (v4.9, 2-20-2017)[11] was used for motif analysis and scanning.

**Figure legends**

**Supplementary Figure 1. DUX4-Responsive-Element and electron density map. a)** The consensus DRE sites derived from leukemia NALM6 cells[3]. The ERG binding site (forward chain, 5’-TGATGAGATTA-3’; reverse chain, 3’-ACTACTCTAAT-5’) uncovered by Zhang and co-workers[4] are shown in blue. The GAT repeats in the forward chain are highlighted with red bars above the sequences. The TAAT repeats in the reverse chain are highlighted with purple bars below the sequences. **B)** 2*Fo*-*Fc* electron density map of DUX_HD2_-DNA_ERG_ at 1 σ level. The refined model is shown in stick representation.

**Supplementary Figure 2. DUX4 HDs in homeobox superfamily. a**) Sequence alignment between DUX4_HD2_ and its homeobox homologs. The poly-Arg/Lys and QNR motifs are colored in green and red, respectively. Additional DNA binding residues in DUX4, PAX, PAX3 are colored in blue. **b**) Summary of HD-DNA_motif_ interaction. Of note, until this report is written, DUX4 HD1-HD2 (red) is the only double homeobox that has dual preference over TGAT- and TAAT-like repeats.

**REFERENCEs**

9. Eidahl JO, Giesige CR, Domire JS, Wallace LM, Fowler AM, Guckes S*, et al.* Mouse Dux is myotoxic and shares partial functional homology with its human paralog DUX4. *Human Molecular Genetics* 2016; **25**(20)**:** ddw287.

10. Quinlan AR, Hall IM. BEDTools: a flexible suite of utilities for comparing genomic features. *Bioinformatics* 2010; **26**(6)**:** 841.

11. Heinz S, Benner C, Spann N, Bertolino E, Lin YC, Laslo P*, et al.* Simple Combinations of Lineage-Determining Transcription Factors Prime -Regulatory Elements Required for Macrophage and B Cell Identities. *Molecular Cell* 2010; **38**(4)**:** 576.
