# Supplementary figures and images for: DUX4HD2-DNAERG structure reveals new insight into DUX4-Responsive-Element
Source: Leukemia. 2018 Oct 12;33(2):550–3. doi: 10.1038/s41375-018-0273-z (PMC6365376; doi:10.1038/s41375-018-0273-z)

# Supplementary Figure 1 Dong et al, 2018

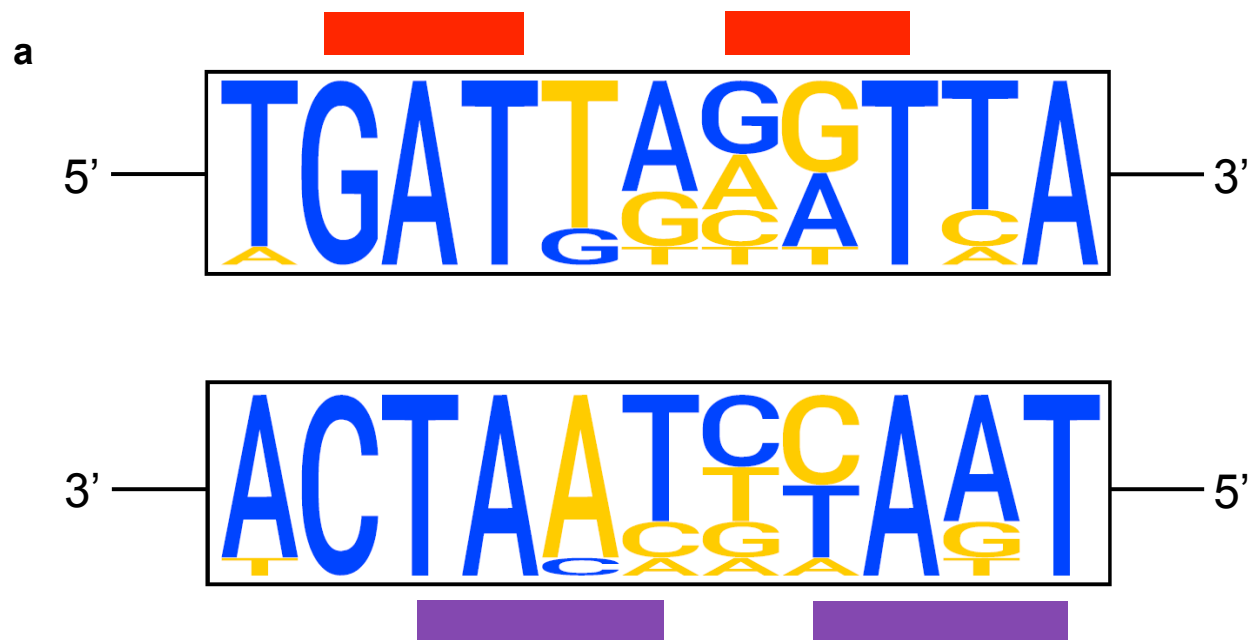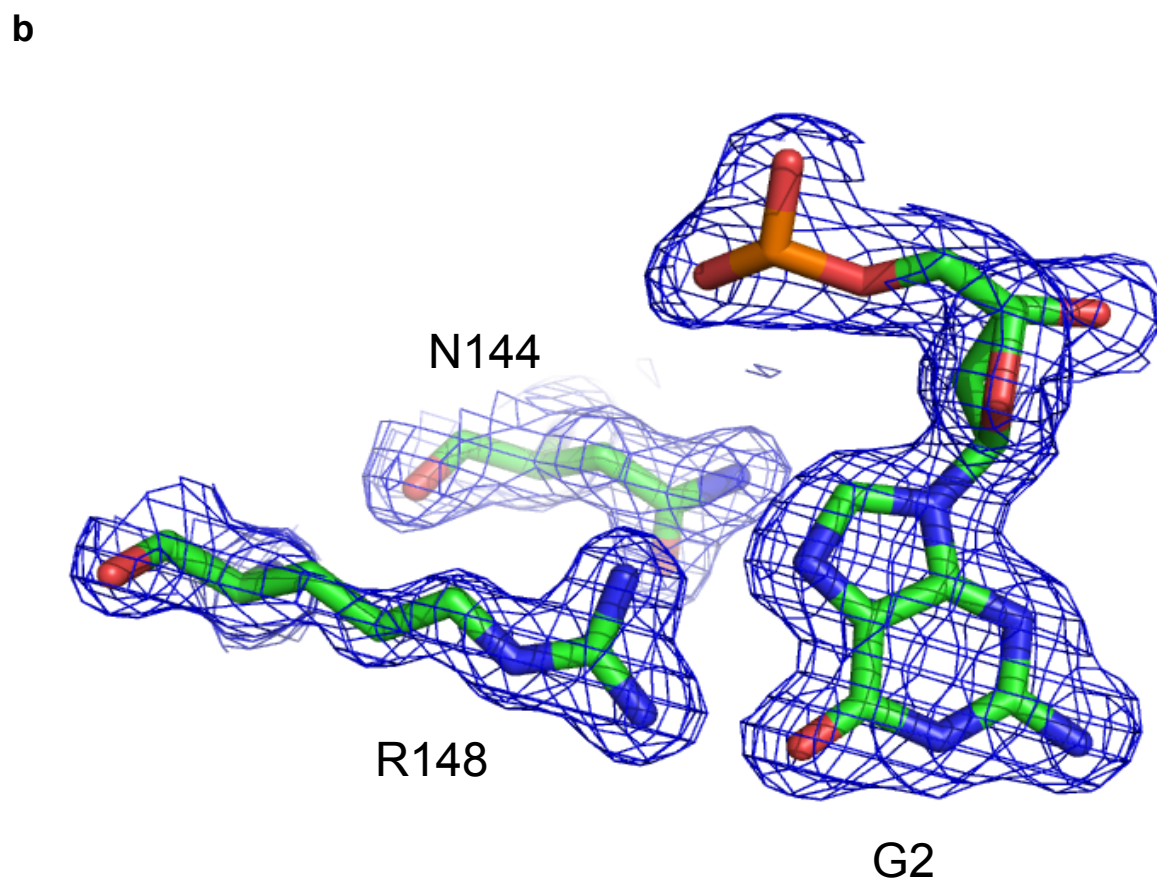

Supplement: Supplementary file 2 — Supplementary Figure 1 [file 41375_2018_273_MOESM2_ESM.pdf]
