# Supplementary material for: DUX4HD2-DNAERG structure reveals new insight into DUX4-Responsive-Element
Source: Leukemia. 2018 Oct 12;33(2):550–3. doi: 10.1038/s41375-018-0273-z (PMC6365376; doi:10.1038/s41375-018-0273-z)
Supplement: Supplementary file 3 — Supplementary Figure 2 [file 41375_2018_273_MOESM3_ESM.pdf]

Supplementary Figure 2 Dong et al, 2018

a

```
          94        103       113       133       143
DUX4HD2   GRRKRTAVTGSQTALLLRAF---LARETGLPESRIQIWFQNRRARHP
DUX4HD1   GRRRRLVWTPSQSEALRACF---LAQAIGIPEPRVQIWFQNERSRQL
Pax       QRRSRTTFSASQLDELERAF---LAQRTNLTEARIQVWFQNRRARLR
Pax3      QRRSRTTFTAEQLEELERAF---LAQRAKLTEARVQVWFSNRRARWR
ExdPA     ARRKRRNFSKQASEILNEYF---LARKCGITVSQVSNWFGNKRIRYK
Six1      GEETSYCFKEKSRGVLREWY---LAEATGLTTTQVSNWFKNRRQRDR
Pit1      KRKRRTTISIAAKDALERHF---MAEELNLEKEVVRVWFCNRRQREK
PBX1      ARRKRRNFNKQATEILNEYF---LAKKSGITVSQVSNWFGNKRIRYK
Pou1F1    KRKRRTTISIAAKDALERHF---MAEELNLEKEVVRVWFCNRRQREK
Mata1     SPKGKSSISPQARAFLEEVF---VAKKCGITPLQVRVWFINKRMRSK
Bicoid    PRRTRTTFTSSQIAELEQHF---LSAKLALGTAQVKIWFKNRRRRHK
Pax6      GQRNRTSFTQEQIEALEKEF---LAAKIDLPEARIQVWFSNRRAKWR
Pitx2     QRRQRTHFTSQQLQELEATF---IAVWTNLTEARVRVWFKNRRAKWR
HoxB1     PSGLRTNFTTRQLTELEKEF---IAATLELNETQVKIWFQNRRMKQK
Lhx4      AKRPRTTITAKQLETLKNAY---LSSETGLDMRVVQVWFQNRRAKEK
Aris      QRRYRTTFTSFQLEELEKAF---LAMKIGLTEARIQVWFQNRRAKWR
UbxPB     RRRGRQTYTRYQTLELEKEF---MAHALCLTERQIKIWFQNRRMKLK
HoxA9     TRKKRCPYTKHQTLELEKEF---VARLLNLTERQVKIWFQNRRMKMK
HoxA13    GRKKRVPYTKVQLKELEREY---ISATTNLSERQVTIWFQNRRVKEK
HoxB13    ERKKRIPYSKGQLRELEREY---ISAATSLSERQITIWFQNRRVKEK
CDX1      KDKYRVVYTDHQRLELEKEF---LAANLGLTERQVKIWFQNRRAKER
Msx1      NRKPRTPFTTAQLLALERKF---FSSSLSLTETQVKIWFQNRRAKAK
Dlx5      VRKPRTIYSSFQLAALQRRF---LAASLGLTQTQVKIWFQNKRSKIK
AntpHD    RKRGRQTYTRYQTLELEKEF---IAHALSLTERQIKIWFQNRRMKWK
Gbx1      SRRRRTAFTSEQLLELEKEF---IAHALKLSEVQVKIWFQNRRAKWK
ScrExd    TKRQRTSYTRYQTLELEKEF---IAHALSLTERQIKIWFQNRRMKWK
Nkx2.5    RRKPRVLFSQAQVYELERRF---LASVLKLTSTQVKIWFQNRRYKSK
                  .      *   :   .:    :    :  ** *.* :
```

b

Motif	Protin
TAAT	$DUX4_{HD2}$
TAAT	Pax
TAAT	Pax3
TAAT	Bicoid
TAAT	Pitx2
TAAT	Aristaless
TAAT	Msx1
TAAT	Dlx5
TAAT	AntPHD
TAAT	Gbx1
TAAT	Lhx4
TTAT	SrcExd
TTAT	UbxPB
TTAT	HoxA13
TTAT	HoxB13
TGAT	$DUX4_{HD2}$
TGAT	ExdPA
TGAT	PBX1
TGAT	Mata1
TGAT	HoxB1
TCAT	Pou1F1
TTAC	HoxA9
TAGA	CDX1
CAAG	Nkx2.5
ACAT	Pit1
